# Acceptance, utilization, and factors associated with immediate postpartum intrauterine contraceptive device among mothers delivered at public health facilities in Hawassa city, Ethiopia: Institution-based study

**DOI:** 10.1186/s12978-023-01586-z

**Published:** 2023-03-08

**Authors:** Yemisrach Shiferaw, Meskerem Jisso, Selam Fantahun, Betelhem Eshetu, Abiyu Ayalew Assefa, Achamyelesh Gebretsadik

**Affiliations:** 1 Hawassa University College of Medicine and Health Science School of Public Health, Hawassa, Ethiopia; 2 Department of Public Health, Hawassa College of Health Science, P. O. Box: 84, Hawassa, Ethiopia

**Keywords:** Acceptance, Ethiopia, Hawassa, Immediate postpartum, Intrauterine contraceptive device, Utilization

## Abstract

**Background:**

Immediate postpartum intra-uterine contraceptive device (IPPIUCD) placement within 10 min after the expulsion of the placenta following vaginal delivery is a safe and effective method when provided after comprehensive counseling. Studies on its acceptance and utilization are scarce in the study area. This study aims to assess the acceptance and utilization of IPPIUCD.

**Methods:**

A cross-sectional study was conducted from January 1st up to February 31st, 2020, among 392 mothers who delivered at public health facilities in Hawassa city. EPI-Data version 7.2 was used for data entry and STATA 14 for analysis. Data were collected using an interviewer administered structured questionnaire. A binary logistic regression and a multivariable logistic regression model were used to assess association. Statistical significance was determined at a p-value of less than 0.05 with a 95% confidence interval.

**Results:**

Of the 392 mothers enrolled, 16.3% (95% CI: 12.7–20.0) of them accepted immediate post-partum IUCD. However, only 10% (95%CI: 7.0, 12.9) utilized immediate post-partum IUCD. Counseling about IPPIUCD, Attitude, plan to have another child, and birth intervals were associated with acceptance of immediate PPIUCD while husband support for family planning use, delivery time, and the number of children had a significant association with utilization of immediate PPIUCD.

**Conclusions:**

The study found a relatively low proportion of acceptors and utilizers of immediate post-partum IUCD in the study area. To improve the acceptance and utilization of immediate PPIUCD among mothers, all stakeholders concerned with family planning need to mitigate and promote the challenges and facilitating factors, respectively.

## Plain Language Summary

Immediate post-partum intrauterine contraceptive device (IPPIUCD) is a safe and effecti ve family planning method to prevent unintended pregnancy for a long time, by making the mother protected immediately after delivery before she leaves the postnatal room. Since it was a newly initiated program in Ethiopia, the proportion of mothers accepting and utilizing IPPIUCD and what factors should be the factors to acceptance and utilization were not well known. Previous studies were either from sole facility data or focused on postnatal family planning in general. This study was intended to assess acceptance, utilization, and related factors about IPPIUCD in 392 women delivered in five public health facilities. The facilities range from health centers to general and referral hospitals, providing immediate post-partum IUCD, free of charge without the need of returning for family planning methods. The proportion of women who accepted and utilized IPPIUCD was very low. Receiving information about IPPIUCD through counseling and a favorable attitude about IUCD resulted in better acceptance of PPICD than not being counseled and having an unfavorable attitude. Additionally, mothers with short birth intervals, due to fear of early pregnancy and wanting to limit pregnancy due to having a sufficient number of children were better acceptance than their counterparts. More utilization was observed among women who gave birth in the daytime, having satisfied the demand for childbirth and husband support for family planning use. To increase acceptance and utilization of IPPIUCD health professionals should provide counseling about IPPIUCD for all women, including their husbands. We have to consider family planning equally in daytime and nighttime services.

## Introduction

Globally, 121 million unintended pregnancies occurred at an annual rate of 64/1000 women [1]. This rate is higher in sub-Saharan Africa 99 per1000 pregnancy and in Ethiopia, 100 per1000 were unintended [2]. Met demand for family planning is one of the indicators in measuring the attainment of Sustainable Development Goal five and a strategy for reducing maternal, infant, and child mortalities by reducing unintended pregnancy [3]. Long-acting reversible contraception and comprehensive contraceptive counseling increase accessibility, have a high continuation rate, and provide greater protection against unintended pregnancy [4, 5]. Intrauterine devices (IUDs) are long-acting reversible contraceptive (LARC) family planning methods in which a couple uses them to limit or space the number of children they want to have through the use of contraceptive methods.

Immediate post-partum intrauterine contraceptive device (IPPIUCD) placement within 10 min after the expulsion of the placenta up to 48 h after delivery is a prevailing strategy that prevents unintended pregnancy with a high continuation rate [6]. Placement of the IPPIUCD is preferred because it does not interfere with breastfeeding and in addition breastfeeding can reduce early removal of IUCD as it reduces bleeding and pain [7, 8]. A short birth interval will be alleviated by using IPPIUD placement [9]. Women who may not return for the postpartum visit [10] and resume sex without using contraception [11], benefit from IPPIUCD. More than half of mothers resume sexual intercourse before 6 weeks postpartum [12, 13]. This is due to the low perception of the risk of pregnancy [12].

Acceptance is important for the utilization of any effective method of family planning. Acceptance and utilization of IPPIUD were higher for those mothers who had third-trimester visits [14]. Counseling is one of the tools to increase the acceptance of IPPIUCD [15]. Counseling about PPIUCD during antenatal care (ANC), spousal approval, having more than one child, and short-interval pregnancy favored the use of IPPIUCD [15]. In Ethiopia, despite several advantages and high effectiveness among the long-acting reversible contraceptives and existing additional opportunities from the increasing rate of institutional delivery for utilization of IPPIUCD, it utilization remains very low at 2% [16, 17]. The acceptance and utilization of IPPIUCD's in Ethiopia, particularly in the study area, is insufficient. Thus, this study aimed to assess the acceptance, utilization, and contributing factors of immediate post-partum IUCD among mothers coming for delivery service in Hawassa city.

## Materials and methods

### Study design, period, and setting

A facility-based cross-sectional study was carried out from January 1st to February 31st of 2020 in Hawassa City which is located 275 km south of Addis Ababa (the capital city of Ethiopia) on the Trans-African Highway 4 Cairo-Cape Town. Hawassa City is serving as the capital city of both Sidama regional state and southern nation national people regional (SNNPR). In Ethiopia including Hawassa city, all family planning methods and counseling services are offered for free. There are 12 public health facilities that provide delivery service in the city.

### Study population

All postnatal mothers who gave birth at public health facilities of Hawassa city were considered as the source population while postnatal mothers who gave birth at selected public health facilities of Hawassa city during the study period were considered as the study population.

### Eligibility criteria

Postnatal mothers who gave birth at immediate PPIUCD providing public health facilities of Hawassa city during the study period and residing for six months in Hawassa City were included. Mothers who were in poor health condition and did not fulfill the world health organization medical eligibility criteria for IUCD insertion were excluded.

### Sample size calculation

The sample size (n) required for this study was determined using single population proportion formula (n = (Zα/2)^2^ p (1 − p)/d^2^)) by considering the following assumptions; the proportion of women who accepted post-partum IUCD use as 38% (p = 0.38) taken from a study conducted at Sidama zone health facilities [18], Za/2 = 1.96 (significance level at α = 0.05 with 95% confidence interval) and margin of error to be 5%. Adding a 10% non-response rate. Thus, a total of 398 sample sizes was obtained.

### Sampling procedure

From all IPPIUCD service providing health facilities in Hawassa City, two hospitals (Hawassa comprehensive referral hospital and Adare general hospital) and three health centers (Adare, Millennium, and Alamura) were selected randomly. Then, the calculated minimum sample size was allotted by proportionate allocation based on a one-year delivery report, after calculating the average estimated monthly delivery in each health facility. Accordingly, a sample of 169, 168, 17, 26, and 18 were allocated for Hawassa comprehensive specialized hospital, Adare general hospital, Adare health center, Millennium health center, and Alamura health centers, respectively. Study participants were selected consecutively from each randomly selected health facility by considering their order of attendance at the delivery service to be random.

### Study variables

Acceptance and utilization of immediate postpartum intrauterine contraceptive device were a dependent variable, while socio-demographic characteristics (age, marital status, place of residence, educational status, occupational status, husband occupational status, husband educational status, religion, and average monthly family income), reproductive health-related characteristics (gestational age, number of children, birth interval, birth plan for other children, future number of children, resumption of sexual and menstrual period following previous delivery), family planning related characteristics (counseling about IPPIUCD, use of F/P in the past, decision for modern contraception, and husband support to use family planning), health service utilization (ANC follow up, mode of delivery, types of delivery), and cognitive related factors (level of knowledge and attitude towards the use of PPIUCD) were independent variables of the study.

### Operational definition

#### Acceptance of IPP IUCD

Woman’s verbal consent to use IUCD within 10 min to 48 h of delivery of placenta during the counseling of PPIUCD [19].

#### Utilization of IPPIUCD

Women who accepted PPIUD as a method of family planning and had actual PPIUCD insertion after the post-placental period or before they were discharged from the health facilities [18].

#### Knowledge

In this study, knowledge about immediate PPIUCD was measured by calculating the mean score of eight knowledge-related items and classifying them as good knowledge if the mother responded correctly greater than or equal to the mean score of knowledge questions or poor knowledge if the woman responded correctly less than the mean score of knowledge questions.

#### Attitude

In this study, attitude towards immediate PPIUCD use was measured by calculating the mean score of five attitude-related items and classifying them as positive attitude if the woman responded correctly greater than or equal to the mean score of attitude questions or negative attitude if the woman responded correctly less than to the mean score of attitude questions.

### Data collection technique and quality

The data were collected using a face-to-face interview technique with a pre-tested structured questionnaire. The questionnaire was developed after reviewing different literatures [12, 13, 18–20]. Initially, the questionnaire was prepared in English and translated into Amharic by an expert, then back to English to check its consistency. Data were collected by five trained midwifery nurses in the postnatal room before discharge. During data collection, every collected questionnaire was checked daily by supervisors and investigators for completeness and consistency.

### Data management and analysis

The collected data were entered checked its validity using EPI-Data version 7.2 and exported to STATA version14 software for editing, cleaning and analysis. Descriptive statistics were used to describe the characteristics of the study respondents by using means and standard deviations for numerical variables, frequencies along with percentages for categorical variables, tables and graphs. Bivariable and multivariable logistic regression analyses were used to identify influencing factors affecting the acceptance and utilization of IPPIUD. All explanatory variables in bivariable analysis with a p-value of less than 0.25 (p-value < 0.25) were selected as candidate variables for multivariable logistic regression analysis. The crude and adjusted odd ratios together with their corresponding 95% confidence interval were computed and interpreted accordingly. In the final model, the adjusted odds ratio (AOR) with their corresponding 95% confidence interval at a p-value of < 0.05 was used to declare a significant association. Hosmer–Lemeshow test was used to compare and rule out the goodness of fit of the models and it was non-significant. A multicollinearity test was conducted among independent variables and it was found not a problem.

## Results

A total of 392 respondents were participated in this study yielding a response rate of 98.5%. the main reason for non-response was due to incomplete data on filled questionnaires and refusals of respondents to take part in our study.

### Socio-demographic characteristics of mothers

The majority of the respondents 142 (36.2%) were within the age group of 20–25 years, with the mean (± SD) age of 25.18 (± 4.56) years. Most of the respondents 382 (97.9%) and 364 (92.9%) were married and urban dwellers, respectively. Half of the respondents were Protestant 223(56.9%) followed by Orthodox Christians 103 (26.3%) by their religion. Regarding their occupational status, 211 (54%) of respondents were housewives while 77 (17.6) were government employees. Of the total respondents, 148(38%) of them completed primary level of education while 141 (36.0%) of their husbands completed college and above level of education. Furthermore, 119 (30.4%) of their husbands were private employees by their occupational status (Table 1).

**Table 1:** Socio-demographic and economic characteristics among mothers delivered at public health facilities in Hawassa city, Ethiopia, 2020

Variables	Frequency	Percent
Age of respondent		
< 20	76	19.4
20–25	142	36.2
26–30	135	34.4
> 30	39	9.9
Educational status of the respondent		
Secondary and below	290	74.0
College and above	102	26.0
Occupational status of respondents		
Housewife	211	53.8
Governmental employee	77	19.6
Merchant/private employee	70	17.9
Student	27	6.9
Others^a^	7	1.8
Marital status		
Married	384	98.0
Single	8	2.04
Place of residence		
Urban	364	92.9
Rural	28	7.1
Average monthly income of the family (ETB)		
0–36,000	163	41.6
36,001–60,000	100	25.5
> 60,001	129	32.9
Religion of respondents		
Protestant	223	56.9
Orthodox	103	26.3
Muslim	58	14.8
Others^b^	8	2.0
Husband occupational status		
Merchant	111	28.3
Governmental employee	105	26.8
Private employee	119	30.4
Others^c^	49	12.1
Not applicable	8	2.0
Husband educational status		
No formal education	24	6.1
Primary school	102	26.0
Secondary school	117	29.8
College and above	141	36.0
Not applicable	8	2.0

*ETB* Ethiopian birr

^a^Others**:** daily labor, farmer

^b^Others: Catholic, Jehovah’s witness

^c^Others: indicate: Daily labor, student, farmer

### Reproductive health-related characteristics of mothers

The mean (± SD) number of living children was 1.77 (± 0.71) per respondent. One hundred eighty-four (46.9%) respondents had one to two children before this delivery while 150 (38.3%) of the hadn’t child. Birth intervals of more than three years were reported by 119 (30.4) respondents. Nearly half, (45.7%) of the respondents had the desire to have four children in the future and 237(67.7%) of respondents had a future birth plan after three years. Of all respondents who had an experience of delivery, 170 (72.6%) and 119 (51.1%) of them had sexual and menstrual resumption after 45 days of their previous delivery, respectively (Table 2).

**Table 2:** Reproductive health characteristics among mothers delivered at public health facilities in Hawassa city, Ethiopia, 2020

Characteristics	Frequency	Percent
Gestational age		
Term	362	92.3
Preterm	23	5.9
Post-term	7	1.8
Number of children		
No child	150	38.3
1–2 Children	186	47.4
≥ 3 Children	56	14.3
Birth interval		
Below 24 months	173	44.1
24–36 months	100	25.5
Above36 months	119	30.4
Birth plan to other children		
Yes	347	88.5
No	45	11.5
Future number of children		
Below 4 children	88	24.2
4 children	179	49.3
Above 4 children	96	26.4
Not decided on the number	29	7.4
Future time to give birth		
Below 24 months	10	2.9
24–36 months	103	29.4
Above 36 months	237	67.7
Timing of resumption of sexual intercourse after a previous delivery		
Before 45 days	47	20.1
After 45 days	170	72.6
After 6 months	17	7.3
Timing of resumption of menstruation after a previous delivery		
Before 45 days	34	14.6
After 45 days	119	51.1
After 6 months	80	34.3

### Family planning-related characteristics of mothers

Of the total respondents, 312 (79.6%) had ever heard about IUCD. The majority of 241 (61.5%) respondents received counseling about IPPIUCD, of whom 160 (66.4%) were counseled during antenatal care (ANC). Most of the respondents 264 (67.7%) used family planning at any time in the past, of them 254 (96.2%) used it before to the current pregnancy. Two hundred one (58%) of respondents decided about modern contraception use with their husbands while 234 (60.9%) of respondents had partner support to use family planning (Table 3).

**Table 3:** Family planning-related history among mothers delivered at public health facilities in Hawassa city, Ethiopia, 2020

Variable	Frequency	Percent
Ever heard of IUCD		
Yes	312	79.6
No	80	20.4
Counseling about IPPIUCD		
Yes	241	61.5
No	151	38.5
Time of counseling		
ANC	160	66.4
Delivery	16	6.6
Postnatal	65	27.0
Use of F/P in the past		
Yes	264	67.7
No	126	32.3
Use F/P before the current pregnancy		
Yes	254	96.2
No	10	3.8
Types of F/P method used before current pregnancy		
Implant	102	40.1
IUCD	23	9.1
Pills	27	10.6
Condom	3	1.2
Injectable	99	39.0
The decision for modern contraception		
Me and my husband	201	58.1
Me	87	25.1
Husband	49	14.2
Health extension workers/health workers	9	2.6
Husband support to use of FP		
Yes	234	60.9
No	150	39.1

### Knowledge and attitude of mothers towards immediate PPIUCD usage

**Fig. 1:**
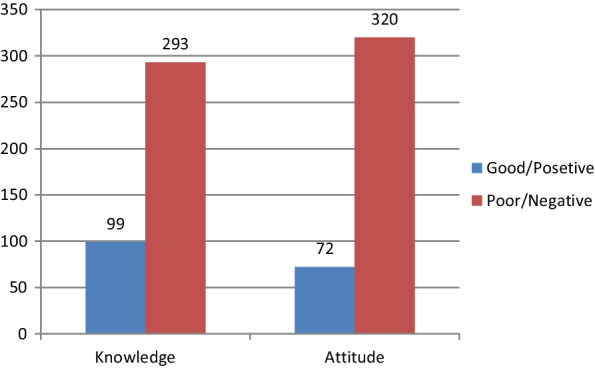
Level of knowledge and attitude towards immediate PPIUCD among mothers delivered at public health facilities in Hawassa city, Ethiopia, 2020

The knowledge mean score of the respondents was 2.6 (± 1.6) SD. About three fourth of respondents (74.7%) had poor knowledge about immediate PPIUCD. Regarding the attitude of mothers toward immediate PPIUCD use, 320 (81.6%) of them had a negative attitude (Fig. 1).

### Health service utilization-related characteristics of mothers

Of the 377 responders, antenatal care attendance was reported by the majority (96.2%), 198 (52.5%) of whom had their fourth ANC visit. Relating to the mode of delivery, about 259 (66.1%) of respondents were delivered through spontaneous vaginal delivery. During the study period, one out of ten current deliveries were unplanned (Table 4).

**Table 4:** Health service utilization among mothers delivered at public health facilities in Hawassa city, Ethiopia, 2020

Characteristics	Frequency	Percent	
ANC follow up			
Yes	377	96.2	
No	15	3.8	
Number of ANC follow up reported n = 377			
1–2 times	47	12.5	
Three times	132	35.0	
Four and above	198	52.5	
Mode of delivery			
Instrumental	36	9.2	
C/S	97	24.7	
Current delivery			
Planed	351	89.5	
Not planned	41	10.5	

### Acceptance and utilization of an immediate PPIUCD

**Fig. 2:**
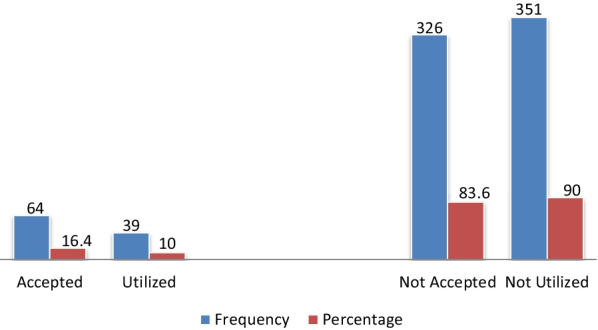
Acceptance and utilization of IPPIUCD among mothers delivered at public health facilities in Hawassa city, Ethiopia, 2020

The overall acceptance of immediate postpartum IUCD as a family planning method was found to be 16.4% (95% CI: 12.8, 20.2) while only 10.0% (95% CI: 7.0, 12.9) of respondents utilized immediate postpartum IUCD as a family planning method (Fig. 2).

### Reasons for not using immediate postpartum IUCD

**Fig. 3:**
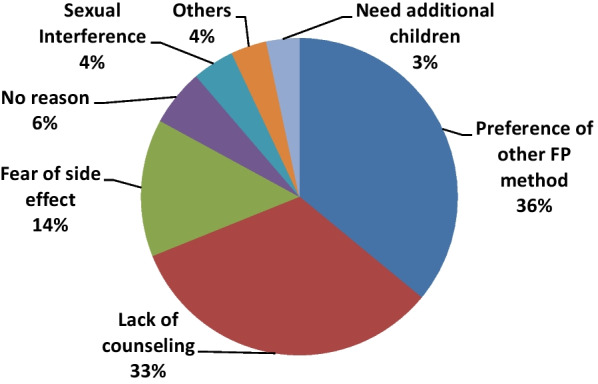
Reason for rejecting IPPIUCD among mothers delivered at public health facilities in Hawassa city, Ethiopia, 2020. *NB* Other; menstrual disturbance, husband refusal, No access

According to the results of this study, the main reason cited for rejecting immediate postpartum IUCD was a preference for another FP method 118 (36%) followed by lack of counseling 108 (32.9) while 19 (5.8%) of women have no reason (Fig. 3).

### Factors associated with acceptance and utilization of immediate post-partum IUCD

The assumptions of logistic regression were checked before conducting a regression analysis. In bivariable logistic regression analysis, seven variables with a p-value of less than 0.25 became eligible for multivariable logistic regression. After controlling for possible confounders in multivariable logistic regression (birth interval, plan to have another child, counseling about IPPIUCD, and attitude towards IUCD) were found to be associated with acceptance of immediate post-partum IUCD.

Accordingly, mothers having less than two years of birth interval increase the odds of acceptance for IPPIUCD by 2.7 times compared with mothers having three or more years of interval (AOR = 2.71; 95% CI (1.09, 6.72). Mothers with a plan to have another child were more than three times more likely to accept IPPIUCD (AOR = 3.32; 95% CI (1.45, 7.59) than mothers who did not have the plan to have another child. The likelihood of accepting IPPIUCD among mothers counseled about it was 3.79 times higher than among mothers not counseled about it (AOR = 3.79; 95% CI (1.67, 8.55), compared to their counterparts. Similarly, the odds of accepting IPPIUCD among mothers having a favorable attitude toward IUCD were six times higher than those of mothers with an unfavorable attitude toward IUCD (AOR = 6.43; 95% CI (3.26, 12.68) (Table 5).

**Table 5:** Factors associated with acceptance of immediate PPIUCD among mothers delivered at public health facilities in Hawassa city, Ethiopia, 2020

Variables	Acceptance		COR (95%CI)	AOR (95%CI)
	Yes	No		
Age (year)				
< 20	7	69	1	1
20–25	15	127	1.16 (0.45, 2.99)	1.02 (0.36, 2.91)
26–30	32	103	3.06 (1.27, 7.33)	2.00 (0.71, 5.66)
> 30	10	29	3.39 (1.17, 9.79)	2.38 (0.66, 8.54)
Birth interval				
Below 24 months	21	152	0.72 (0.37, 1.42)	2.71 (1.09, 6.72)*
24–36 month	24	76	1.66 (0.84, 3.25)	1.86 (0.85, 4.08)
Above 36 months	19	100	1	1
Use of FP in the past				
Yes	53	212	2.63 (1.32, 5.24)	2.00 (0.83, 4.83)
No	11	116	1	1
Plan to have another child				
Yes	50	297	1	1
No	14	31	2.68 (1.33, 5.39)	3.32 (1.45, 7.59)*
Ever heard of IUCD				
Yes	60	252	4.52 (1.59, 12.85)	2.30 (0.75, 7.04)
No	4	76	1	1
Attitude toward immediate PPIUCD				
Favorable	31	39	6.96 (3.84, 12.60)	6.43 (3.26, 12.68)*
Unfavorable	33	289	1	1
Counseling about IPPIUCD				
Yes	55	186	4.66 (2.23, 9.75)	3.79 (1.67, 8.55)*
No	9	142	1	1

*COR* crude odd ratio, *AOR* adjusted odd ratio, *CI* confidence interval, *1* Reference

*Remained significant at P-Value < 0.05

Furthermore, in this study, husband support for family planning use, delivery time, and number of children had significant associations with the utilization of IPPIUCD. Accordingly, mothers who had husband support for family planning use were 3.28 times more likely to utilize IPPIUCD (AOR = 3.28; 95% CI (1.28, 8.41)) than their counterparts. Having delivery during the day increases the odds of utilizing IPPIUCD by 2.25 times (AOR = 2.25; 95% CI (1.06, 4.80) compared with mothers delivering during the night. Similarly, mothers who have more than three children are four times more likely to utilize IPPIUCD (AOR = 4.47; 95% CI (1.43, 13.91)) than those mothers with no children before this birth (Table 6).

**Table 6:** Factors associated with utilization of immediate PPIUCD among mothers delivered at public health facilities in Hawassa city, Ethiopia, 2020

Variables	Utilization		COR (95%CI)	AOR (95%CI)
	Yes	No		
Educational status				
Secondary and below	32	258	1	1
College and above	7	95	0.59 (0.25, 1.39)	0.80 (0.31, 2.05)
Place of residence				
Urban	34	330	0.47 (0.16, 1.32)	1.08 (0.25, 4.60)
Rural	5	23	1	1
Number of children				
No child	10	140	1	1
1–2	18	168	1.50 (0.67, 3.35)	1.88 (0.72, 4.90)
≥ 3	11	45	3.42 (1.36, 8.58)	4.47 (1.43, 13.91)*
Number of ANC follow up				
1–2 times	5	6	1	1
Three times	19	36	1.41 (0.49, 4.02)	1.16 (0.35, 3.89)
Four and above	11	113	0.49 (0.16, 1.49)	0.30 (0.08, 1.13)
Plan to have another child				
Yes	30	317	2.64 (1.16, 6.00)	0.87 (0.27, 2.77)
No	9	36	1	1
Husband support to use FP				
Yes	28	206	2.41 (1.06, 5.44)	3.28 (1.28, 8.41)*
No	8	142	1	1
Time of delivery				
Day	22	136	2.06 (1.05, 4.02)	2.25 (1.06, 4.80)*
Night	17	217	1	1

*COR* crude odd ratio, *AOR* adjusted odd ratio, *CI* confidence interval, *1* reference

*Remained significant at P-Value < 0.05

## Discussion

Immediate postpartum IUCD use is an important approach to avoid unintended pregnancy and improve birth spacing. Hence, this study aimed to assess acceptance, utilization, and factors associated with immediate postpartum intrauterine contraceptive devices among mothers delivered at public health facilities in Hawassa city.

In this study, acceptance of immediate post-partum IUCD was found to be 16.4%. This finding is in line with previous studies conducted in Kenya [21] and the Bale Zone of Ethiopia [19], which reported magnitudes of 12% and 12.4%, respectively. This finding was also found to be low as compared to the studies done in India [22], Egypt [23], Rwanda [24], the Gamo Zone [25], and Sidama Zone [18], which reported acceptance rates of 36%, 28.9%, 67.8%, 35.6%, and 38.1%, respectively. The possible justification could be due to the difference in sample size and level of awareness among mothers. However, this finding was higher than the magnitude of acceptance reported in India [26] which was 8.6%. The possible reason might be due to differences in the study setting and interview period, as most of the women in India were interviewed in the antenatal period.

Results from this study provide an important understanding of the associated risk factors of acceptance of immediate PPIUCD. Accordingly, counseling about IPPIUCD, attitude towards PPIUCD usage, plan to have another child, and birth interval emerged as being independently associated with acceptance of immediate PPIUCD.

According to the current study, mothers who received IPPIUCD counseling had a four times higher likelihood of accepting IPPIUD than mothers who did not. This association was in agreement with a previous study conducted in four countries (India, Nepal, Sri Lanka, and Tanzania) [15], Indonesia [27], India [28], and Gamo Zone, Ethiopia [25]. The possible reason might be that counseling may increase knowledge about PPIUCD and improve the decision-making power of mothers. Supporting evidence is also shown by a study conducted in Pakistan [29] and Rwanda [4], where counseling for mothers during prenatal visits increased the awareness of mothers regarding the PPIUCD. It could also be explained during counseling, when health care providers may clarify misconceptions about PPIUCD and motivate the women to accept PPIUCD immediately after delivery.

The present study indicated that mothers with favorable attitudes were six times more likely to accept IPPIUCD compared with mothers with an unfavorable attitude. This goes with the previous evidence from Nepal [30] and Mekelle City, Ethiopia [31] that reported supportive attitudes as predictors of the acceptability of IUCD. This might be due to having a favorable attitude that may shape the mother's intention for postpartum IUCD willingness.

This study also showed that mothers having a birth interval of below 24 months increase the odds of accepting immediate PPIUCD by 2.7 when compared with mothers having a birth interval of above 36 months. This association agreed with previous studies conducted in Pakistan [29] and Tanzania [32] that found a greater likelihood of post-partum IUCD acceptance if there had been less than two years since the last birth. Additionally, this finding is supported by a study conducted in Rwanda [4, 24] that reports that fear of having an early or unwanted pregnancy was a reason for acceptance of immediate PPFP in 79% of respondents. This could be because mothers who had a short birth interval might require a long-acting and reliable method of contraception to attain optimal birth spacing and nurture their children. According to this finding, it is better to give due attention to a mother with a short birth interval (below 24 months).

Furthermore, the results of this study demonstrate that mothers who do not have a plan to have another child were three times more likely to accept immediate PPIUD than mothers who had a plan to have another child. This finding is supported by a study conducted in the Sidama zone [18], which showed increased use of immediate PPIUCD in mothers who do not have the plan to have another child than in mothers who had the plan to have another child. The possible reason might be that a mother without a plan to have additional children might have enough children, need enough time to recover from the physical stress of one pregnancy before moving on to the next, and have enough time for lactation, which prompted her to accept safe and effective long-acting immediate PPIUCD.

In our study, only 10% (95% CI: 7.0, 12.9) utilized immediate postpartum IUCD as a family planning method. This finding was in line with previous studies conducted in Chamblee, USA [8], 11.7%; India [33], 9.1%; Dila Town [34], 8.2%; and as low as related to magnitudes evidenced in China [35], 14.9%, Rwanda [4], 28.1%; Addis Ababa [37], 26.6%; Gamo Zone [25], 14%; and Sidama Zone [18], 21.9%. However, it was higher than the utilization reported in Kenya [21], Debretabor, Ethiopia [39], and Gojam, Ethiopia [40], which indicated magnitudes of immediate PPIUCD utilization of 5.1%, 1.65%, and 4.02%, respectively. The possible explanation for this discrepancy is variation in the integration of family planning with maternal and child-care services, geographic, socio-demographic, and reproductive characteristics. Furthermore, husband support for family planning use, delivery time, and number of children were significantly associated with utilization of immediate PPIUCD.

This study revealed that mothers with more than three children were above four times more likely to utilize immediate PPIUCD than their counterparts. Similar findings were reported by a study conducted in China [35], Nigeria [41], Rwanda [4, 21], and other parts of Ethiopia [34, 42, 43]. The plausible justification might be due to the fact that mothers who have a large number of children may be inspired to prevent additional pregnancies to limit their family size.

Husband support to use immediate PPIUCD is another variable that remained significantly associated with immediate PPIUCD utilization. Having husband support increases the odds of immediate PPIUCD utilization by 3.28 times compared to mothers lacking husband support. This evidence is comparable with previous studies done in Ghana [44], Addis Ababa [37, 38, 45], and the Gamo Zone (45). This could be a result of the fact that most Ethiopian women do not make decisions about family planning alone. This serves as a reminder that increasing the use of immediate PPIUCD requires incorporating the husband into reproductive health services.

Furthermore, this study indicated that mothers who delivered during the day were 2.25 times more likely to utilize immediate PPIUCD than those who delivered during the night. There is no study with a similar report to support this association, so it needs further study.

Finally, since this study used a cross-sectional design, it is impossible to determine the temporal link between exposure and outcome. Moreover, since the study was carried out in public health facilities, the findings may not adequately reflect the entire population in the city.

## Conclusion

We found low acceptability and utilization of immediate PPIUCD. Counseling about IPPIUCD, attitude, the plan to have another child, and birth Interval were associated with acceptance of immediate PPIUCD, while husband support for family planning use, delivery time, and number of children had a significant association with utilization of immediate PPIUCD.

A low level of acceptability and utilization of immediate PPIUCD can be reduced by routine PPIUD counseling and encouraging couple-based family planning interventions. As a result, the government needs to develop strategies to increase partner involvement in decisions made on reproductive health, family planning in particular.

Finally, determinants of utilization of IPPIUCD are complex and cannot be recognized using quantitative approach only. Therefore, we also recommend conducting of further research using comprehensive qualitative approach to unearth reasons behind the decreased level of IPPIUCD utilization.

## Data Availability

Data is not available for online access, however, readers who wish to gain access to the data can write to the corresponding author Yemisrach Shiferaw at yemisrachshiferaw@yahoo.com.
